# Effectiveness and safety of glucosamine and chondroitin for the treatment of osteoarthritis: a meta-analysis of randomized controlled trials

**DOI:** 10.1186/s13018-018-0871-5

**Published:** 2018-07-06

**Authors:** Xiaoyue Zhu, Lingli Sang, Dandong Wu, Jiesheng Rong, Liying Jiang

**Affiliations:** 1 Baoshan Center for Disease Control and Prevention, Shanghai, People’s Republic of China; 2 Department of Epidemiology, School of Public Health, Nantong University, Nantong, Jiangsu Province People’s Republic of China; 3 Department of Orthopedics Surgery, The Second Affiliated Hospital of Harbin Medical University, Harbin, Heilongjiang Province People’s Republic of China; 4 Shanghai Key Laboratory for Molecular Imaging, Shanghai University of Medicine & Health Sciences, Shanghai, People’s Republic of China

**Keywords:** Osteoarthritis, Glucosamine, Chondroitin, Treatment

## Abstract

**Objective:**

To assess the symptomatic effectiveness and safety of oral symptomatic slow-acting drugs (SYSADOAs) on the treatment of knee and/or hip osteoarthritis, such as chondroitin, glucosamine, and combination treatment with chondroitin plus glucosamine.

**Methods:**

We searched electronic database including PubMed, Embase, Cochrane Library, and the reference lists of relevant articles published from inception to May 22, 2018. An updated meta-analysis was performed to assess the effectiveness of these slow-acting drugs for osteoarthritis.

**Results:**

Twenty-six articles describing 30 trials met our inclusion criteria and were included in the meta-analysis. The estimates between chondroitin and placebo showed that chondroitin could alleviate pain symptoms and improve function. Compared with placebo, glucosamine proved significant effect only on stiffness improvement. However, the combination therapy did not have enough evidence to be superior to placebo. Additionally, there was no significant difference in the incidence of AEs and discontinuations of AEs when compared with placebo.

**Conclusions:**

Given the effectiveness of these symptomatic slow-acting drugs, oral chondroitin is more effective than placebo on relieving pain and improving physical function. Glucosamine showed effect on stiffness outcome. Regarding on the limited number of combination therapy, further studies need to investigate the accurate effectiveness. This information accompanied with the tolerability and economic costs of included treatments would be conducive to making decisions for clinicians.

**Electronic supplementary material:**

The online version of this article (10.1186/s13018-018-0871-5) contains supplementary material, which is available to authorized users.

## Background

Osteoarthritis (OA), characterized by progressive cartilage matrix degradation, subchondral bone sclerosis, and osteophyte formation, is the most common form of arthritis [1, 2]. Globally, the prevalence of OA, particularly of the large weight-bearing joints such as the knee and hip, is also predicted to grow [3]. Presently, OA has emerged as one of the major public health concerns and continues to affect about 10% of men and 18% of women over 60 years of age [1].

Previous studies suggest that aging, genetic predisposition, obesity, inflammation, and excessive mechanical loading predispose to OA occurrence and development [4]. The structural changes result in joint pain and stiffness, swelling, and tenderness, which can eventually lead to disability and affect the quality of life of patients [5]. Treatment strategies of OA include both non-pharmacological and pharmacological therapies. Among pharmacological therapies, analgesics and non-steroidal anti-inflammatory drugs (NSAIDs) are current treatment options for OA because of their well-established effectiveness. However, they act as symptomatic treatments without offering disease modification of OA, and they are accused for increased risk adverse events, including the gastrointestinal and/or cardiovascular system [6]. For this reason, attention has recently been focused on an ideal treatment, which can improve the clinical symptoms of OA with better tolerability and safety profiles, such as symptomatic slow-acting drugs (SYSADOAs) [7].

Glucosamine and chondroitin, as important medicine in those SYSADOA, are naturally occurring compounds in the body functioning as the principal substrates in the biosynthesis of proteoglycan [8, 9]. It is suggested that glucosamine and chondroitin are both partially absorbed and then reaches the joints, exerting on relieving joint pain and slowing the rate of joint destruction and cartilage loss. They are two main categories of agents potentially or theoretically acting as chondroprotective agents and disease-modifying OA drugs (DMOADs) [8, 10]. The effectiveness based on the result of RCT published in 2013 suggested that consumption of chondroitin for certain dosage has a positive effect on pain relief and function improvement [7]. Recently, a trial conducted in 2017 demonstrated a lack of superiority of chondroitin and glucosamine combination therapy over placebo [11]. Although many studies have shown a significant treatment effect, accompanied with remarkable safety, there is still controversy regarding the effectiveness of these putative DMOADs compared with placebo [7, 11]. International guidelines for the management of OA had given an equivocal recommendation of glucosamine and chondroitin, and they are not recommended according to Osteoarthritis Research Society International (OARSI) guidelines published in 2014 [12].

Therefore, based on existing evidence, a study needs to be updated and critically evaluates the current evidence-based information about the administration of glucosamine and chondroitin for the treatment of knee or hip OA. In our study, a relatively comprehensive meta-analysis was performed to assess the effectiveness and safety of putative DMOADs.

## Methods

### Search strategy

We conducted this meta-analysis following the PRISMA extension statement [13]. We systematically searched electronic database including PubMed, Embase, and Cochrane Library based on logic combination of keywords and text words associated with OA to extract concerned RCTs from inception to May 22, 2018. The Internet-based search used the following terms: “arthritis,” “osteoarthritis,” “OA,” “joint disease,” “glucosamine,” “GH,” “GS,” “chondroitin,” “CH,” “CS,” and the corresponding free terms. The search was restricted to English language and studies of human participants. We then screened reference lists of all obtained articles, including relevant reviews, to avoid missing relevant articles. And, we also searched ClinicalTrials.gov for progressive trials.

### Inclusion and exclusion criteria

Studies were included if they met the following criteria: (1) RCTs; (2) studies about primary hip and/or knee OA patients with clinical and/or radiologic diagnosis; (3) studies covering at least two of the following oral treatments: glucosamine, chondroitin, or the two in combination against placebo; and (4) extractable data reporting the pain, function, stiffness, and the adverse events (AEs) of patients.

The exclusion criteria were as follows: (1) studies of non-randomized and/or uncontrolled trials, (2) treatment methods described unclearly, (3) interventions combined with non-steroidal anti-inflammatory drugs, (4) studies or data reported repeatedly, and (5) trial arms with sub-therapeutic doses (< 1500 mg/day of glucosamine and < 800 mg/day of chondroitin (according to dosage licensed in Europe)) [14].

### Data extraction

Two investigators (X.Y.Z and L.L.S) independently assessed all studies for eligibility and extracted data in accordance with a preconfigured form from each study. Any disagreements were resolved through discussion with a third reviewer (L.Y.J). For each study, patients’ characteristics including mean age, sex, mean duration of symptom, BMI, duration of follow-up, type of outcome (pain, function, stiffness, and AEs), trial design, trial size, details of intervention, treatment duration, and results were individually extracted. Data of intention-to-treat analysis was employed whenever possible.

### Quality assessment

The Cochrane Risk of Bias Tool was used to evaluate the methodological quality of the included studies (version 5.3) [15]. The tool evaluated seven potential risks of bias: random sequence generation, allocation concealment, blinding of participants, blinding of outcome assessment, incomplete outcome data, selective reporting, and other bias. Each item was judged by the following criteria: low risk of bias, uncertain risk of bias, and high risk of bias. Whenever studies included three or more high risk of bias, it was considered as poor methodological quality. Two reviewers (X.Y.Z and L.L.S) checked the profile of each included study independently.

### Outcome measures

The primary outcomes of this meta-analysis were pain intensity, function improvement, and stiffness score from baseline to the end of treatment. The secondary outcome was safety of studies. We preferred to the scale that was recognized to be the highest on the hierarchy of those suggested outcomes when more than one pain scales were given for a trial. Among these scales, global pain has precedence over pain on walking and the Western Ontario and McMaster Universities Osteoarthritis Index (WOMAC) pain subscale [16, 17]. Similarly, the data of function and stiffness was extracted with the same method. If global function score was not reported, the walking disability, function subscale of WOMAC, or Lequesne Index would be applied instead.

The standard mean difference (SMD) was used to calculate the difference between two interventions because different studies assessed the same outcome by employing different scales. SMD expresses the size of the intervention effect in each study relative to the variability observed in that study by dividing the pooled SD of the differences between two interventions [18, 19]. The effect size was transformed back to the different units of the WOMAC Visual Analogue Scale (VAS), the most commonly used scale based on a media pooled SD of 2.5 cm to assess pain on the scale of 0 to 10 cm. A standardized WOMAC function score (0–10) was transformed by SMD, which based on a median pooled SD of 2.1 units. A change of 2 points on the 0–10 scale was interpreted clinically significant improvement [20]. The negative effect size indicated a better treatment effect on pain relief and function improvement.

### Statistical analysis

All results summarized using STATA software (version 13.1, StataCorp, College Station, TX). For continuous outcomes, SMD with 95% credible interval (CI) was used to present the effect size. For counting data, we calculated relative risk (RR) with 95% CI. The heterogeneity between studies was tested using the *Q* statistics. *P* < 0.1 was considered statistically significant. And, *I*^2^ was used to quantify the inconsistency among the potentially disparate sources of studies. A random-effects model was used if *I*^2^ > 50%. A subgroup analysis was conducted because there were different types of SYSADOA. Publication bias was examined through visual inspection of funnel plot asymmetry. A sensitivity analysis was performed to evaluate the effect of each study on the combined effect size by omitting each study.

## Results

### Study selection and characteristics

A flowchart of study search and selection was presented in Additional file 1: Figure S1. We identified 1407 references in our literature search and out of 97 potentially eligible studies, 26 articles describing 30 trials met our inclusion criteria and were included in the meta-analysis [7, 11, 21–44]. All trials were published as full journal articles and all trials used a placebo control. Only two articles compared the effectiveness among glucosamine, chondroitin, and the two in combination with placebo at one time [29, 33]. Therefore, 14 RCTs were employed to assess the effectiveness of oral glucosamine, 12 studies were included in the analysis of oral chondroitin, and 4 trials were used to estimate the effectiveness in the subgroup of the combination of glucosamine and chondroitin. Characteristics of included studies were shown in Table 1. All of these included studies were published in English language. A total of 7172 participants were enrolled in this meta-analysis for the pain outcome. Most trials included patients with only knee OA, 1 trial [42] included patients with knee or the hip OA, and 1 trial [31] included patients with the only hip OA. The average age of the patients ranged between 42.65 and 67.09 years (median, 62.28 years), and the percentage of women ranged from 28 to 93% (median, 65%). The average duration of symptoms was reported in 14 trials [11, 21–25, 27–32, 41, 42] and ranged from 1.60 years to 12.98 years (median, 8.05 years).

**Table 1:** Characteristics of the included studies for osteoarthritis of knee and/or hip

Study, year	Treatment (daily dose)	Participants randomized (*n*)	Treatment duration (weeks)	Symptom duration (year)	Mean age (year)	Female (%)	OA grade	Joint	Pain outcome extracted	Timepoint extracted (weeks)
Glucosamine vs placebo										
Noack 1994 [21]	G(1500 mg)/placebo	126/126	1–4	2.00–10.00	55.00	60	I–III	Knee	Lequesne global scale	4
Houpt 1999 [22]	G(1500 mg)/placebo	58/60	1–8	8.30	64.46	62	NA	Knee	WOMAC	12
Reginster 2001 [23]	G(1500 mg)/placebo	106/106	1–144	7.80	65.75	76	II–III	Knee	WOMAC	144
Pavelka 2002 [24]	G(1500 mg)/placebo	101/101	1–144	10.55	62.35	79	II–III	Knee	WOMAC	144
Braham 2003 [25]	G(1500 mg)/placebo	24/22	1–12	12.98	42.65	28	I–III	Knee	KPS	12
McAlinton 2004 [26]	G(1500 mg)/placebo	101/104	1–12	NA	> 65.00	64	NA	Knee	WOMAC	12
Cibere 2004 [27]	G(1500 mg)/placebo	71/66	1–24	1.60	64.48	56	≥ 2	Knee	WOMAC	24
Usha 2004 [28]	G(1500 mg)/placebo	30/28	1–12	3.05	51.03	NA	I–III	Knee	VAS	12
Clegg 2006 [29]	G(1500 mg)/placebo	317/313	1–24	9.95	58.40	63	II–III	Knee	WOMAC	24
Herrero-Beaumont 2007 [30]	G(1500 mg)/placebo	106/104	1–12	7.30	63.94	93	II–III	Knee	WOMAC	24
Rozendaal 2008 [31]	G(1500 mg)/placebo	111/111	1–12	11.70	63.40	69	> 2	Hip	WOMAC	96
Giordano 2009 [32]	G(1500 mg)/placebo	30/30	1–12	6.30	57.65	70	I–III	Knee	WOMAC	24
Fransen 2014 [33]	G(1500 mg)/placebo	152/151	1–48	> 2.00	60.90	83	NA	Knee	WOMAC	96
Kwoh 2014 [34]	G(1501 mg)/placebo	98/103	1–12	NA	52.23	49	0–4	Knee	WOMAC	24
Chondroitin vs Placebo										
Bucsi 1998 [35]	C(1200 mg)/placebo	39/46	1–12	> 0.50	59.95	60	I–III	Knee	VAS	24
Bourgeois 1998 [36]	C(1200 mg)/placebo	83/44	1–13	NA	63.35	76	I–III	Knee	VAS	13
Uebelhart 1998 [37]	C(1200 mg)/placebo	23/23	1–48	NA	58.50	52	I–III	Knee	VAS	48
Mazieres 2001 [38]	C(1200 mg)/placebo	63/67	1–12	NA	67.09	75	II–III	Knee	Pain at rest	12
Uebelhart 2004 [39]	C(1200 mg)/placebo	54/56	1–12	NA	63.45	81	I–III	Knee	Husskisson visual analogue score for pain	12
Michel 2005 [40]	C(1200 mg)/placebo	150/150	1–96	NA	62.80	51	I–III	Knee	WOMAC	96
Clegg 2006 [29]	C(1200 mg)/placebo	318/313	1–24	9.60	58.20	64	II–III	Knee	WOMAC	24
Mazieres 2006 [41]	C(1200 mg)/placebo	153/154	1–24	6.40	66.00	70	II–III	Knee	Pain at rest	24
Kahan 2009 [42]	C(1200 mg)/placebo	309/313	1–12	6.30	62.30	68	I–III	Knee/hip	WOMAC	12
Wildi 2011 [43]	C(800 mg)/placebo	35/34	1–48	> 0.50	62.26	59	I–III	Knee	WOMAC	48
Zegels 2013 [7]	C(1200 mg)/placebo	236/117	1–12	NA	65.17	65	NA	Knee	Global pain	12
Fransen 2014 [33]	C(800 mg)/placebo	151/151	1–48	> 2.00	60.05	83	NA	Knee	WOMAC	96
Glucosamine + Chondroitin vs Placebo										
Clegg 2006 [29]	G + C(1500 + 1200 mg)/placebo	317/313	1–24	9.80	58.40	63	II–III	Knee	WOMAC	24
Fransen 2014 [33]	G + C(1500 + 800 mg)/placebo	151/151	1–48	> 2.00	60.65	85	NA	Knee	WOMAC	96
Lugo 2016 [44]	G + C(1500 + 1200 mg)/placebo	65/58	1–12	NA	52.84	54	II–III	Knee	WOMAC	24
Roman-Blas 2017 [11]	G + C(1500 + 1200 mg)/placebo	80/78	1–24	6.20	65.99	84	II–III	Knee	Global pain	24

*G* glucosamine, *C* chondroitin, *G + C* glucosamine + chondroitin, *NA* not available, *WOMAC* Western Ontario and McMaster Universities, *KPS* Knee Pain Scale, *VAS* Visual Analogue Scale

### Risk of bias

Risk of bias in those included studies was summarized in Additional file 1: Figure S2. All studies were judged as low risk of bias for blinding to patients. Randomization was mentioned in all trails. Nevertheless, 6% did not report details of adequate sequence generation. All studies were judged as low risk of bias for blinding for patients, while 65% for blinding to outcome assessment. In addition, 15% trails did not describe the method of allocation concealment and 92% reported complete outcome data. None of the studies was thought to have poor methodological quality.

### Pain

All studies (7127 patients) contributed to the meta-analysis of pain-related outcomes for the putative DMOADs compared with placebo (Table 1). Fourteen trials (2845 randomized patients) compared glucosamine with placebo [21–34]. Twelve trials (3082 randomized patients) compared chondroitin with placebo [7, 29, 33, 35–43]. Four trials (1200 randomized patients) compared the two in combination with placebo [11, 29, 33, 44].

The meta-analysis identified an overall effect size of − 0.071 (95% CI, − 0.228 to 0.085). When the SMD was transformed, glucosamine showed no significant effect compared with placebo (effect size, − 0.263 cm [95% CI, − 0.635 to 0.113 cm]). However, chondroitin showed better effect compared with placebo (effect size, − 0.540 cm [95% CI, − 0.900 to − 0.178 cm]). Glucosamine plus chondroitin presented no significant effect when compared with placebo (effect size, 1.980 cm [95% CI, − 0.740 to 4.700 cm]) (Table 2). A funnel plot based on studies on the effect size was generated to detect the potential publication bias, and it manifested a significant asymmetry in Additional file 1: Figure S3.

**Table 2:** Effect sizes of symptomatic outcomes

Outcomes	Interventions	No. of studies	Test of association			Test of heterogeneity		
			SMD	95% CI	*P* value	Model	*I*^2^ (%)	*P* value
Pain	G vs. PBO	14	− 0.105	(− 0.254, 0.045)	0.170	Random	72.50	0.000
	C vs. PBO	12	− 0.216	(− 0.360, − 0.071)	0.003	Random	70.80	0.000
	G + C vs. PBO	4	0.792	(− 0.296, 1.880)	0.153	Random	98.50	0.000
	Overall	30	− 0.071	(− 0.228, 0.085)	0.369	Random	90.10	0.000
Function	G vs. PBO	11	− 0.126	(− 0.264, 0.012)	0.073	Random	64.10	0.002
	C vs. PBO	10	− 0.220	(− 0.358, − 0.081)	0.002	Random	68.30	0.001
	G + C vs. PBO	4	0.556	(− 0.368, 1.480)	0.238	Random	98.00	0.000
	Overall	25	− 0.090	(− 0.242, 0.061)	0.242	Random	89.00	0.000
Stiffness	G vs. PBO	8	− 0.305	(− 0.609, − 0.002)	0.048	Random	89.00	0.000
	C vs. PBO	3	0.026	(− 0.073, 0.126)	0.604	Fixed	31.70	0.232
	G + C vs. PBO	2	− 0.070	(− 0.214, 0.074)	0.340	Fixed	0.00	0.582
	Overall	13	− 0.142	(− 0.301, 0.017)	0.081	Random	82.90	0.000

*G* glucosamine, *C* chondroitin, *G + C* glucosamine + chondroitin, *PBO* placebo

### Function

Twenty-five trials (6667 patients) contributed to the meta-analysis of physical function. Table 2 showed estimates across different treatments compared with placebo. In general, the summary of DMOADs had a better effect compared with placebo. The overall effect size was − 0.090 (95% CI, − 0.242 to 0.061). After being transformed, the effect size for the subgroup of chondroitin versus placebo was − 0.462 units (95% CI, − 0.752 to − 0.170 units). Meanwhile, other comparisons presented no significant effect.

### Stiffness

Thirteen trials (4079 patients) contributed to the outcome of stiffness. The overall difference in stiffness improvement versus placebo was − 0.142 (95% CI, − 0.301 to 0.017) for the summary of these treatments, − 0.305 (95% CI, − 0.609 to 0.002) for glucosamine, 0.026 (95% CI, − 0.073 to 0.126) for chondroitin and − 0.070 (95% CI, − 0.214 to 0.074) for the combination of glucosamine and chondroitin (Table 2). In terms of stiffness, only glucosamine showed statistical significance when compared with placebo.

### Safety

**Fig. 1:**
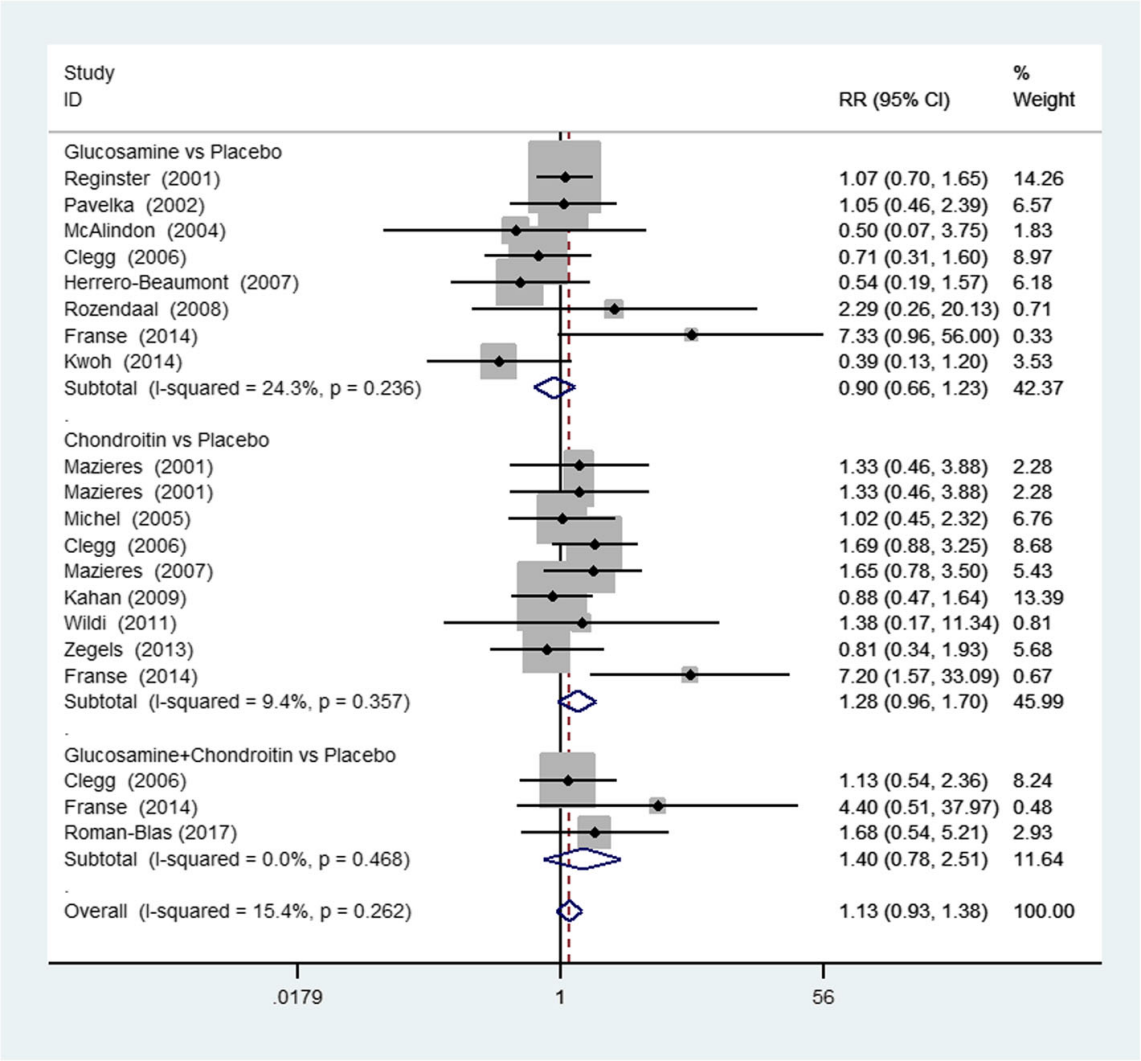
Forest plot of RR and 95% CIs of studies of adverse events. RR relative risk, 95% CI confidence interval, G + C glucosamine + chondroitin

Twenty studies reported the withdrawals of patients due to AEs. Eight studies reported the number of patients with AEs such as diarrhea, abdominal pain, nausea, headache, and others. Figure 1 showed the results of safety and tolerability including the number of withdrawals due to AEs. There was no significant difference in the comparison between any options versus placebo. In addition, six specific kinds of AEs were also analyzed by meta-analysis, and the results were presented in Table 3. The meta-analysis of those studies showed that there was no statistically significant difference between the group of SYSADOAs and placebo group.

**Table 3:** Risk ratio (95% CI) of specific adverse effects between different treatment groups

Comparison	GI AE	CV AE	CNS AE	MU AE	Infection	Skin AE	Others
G vs PBO	0.99(0.79, 1.23)	NA	0.72(0.46, 1.10)	1.52(0.88, 2.63)	1.07(0.50, 2.32)	0.80(0.38, 1.68)	1.21(0.98, 1.48)
C vs PBO	0.35(0.14, 0.87)	1.13(0.45, 2.84)	0.79(0.37, 1.67)	NA	0.98(0.72, 1.34)	1.00(0.41, 2.45)	NA
G + C vs PBO	2.79(0.30, 26.00)	NA	1.86(0.36, 9.74)	2.79(0.30, 26.00)	2.79(0.12, 67.10)	NA	4.66(0.23, 94.79)
Overall	0.92(0.74, 1.13)	1.13(0.45, 2.84)	0.77(0.54, 1.11)	1.58(0.93, 2.70)	1.01(0.76, 1.35)	0.88(0.50, 1.55)	1.22(1.00, 1.50)

*G* glucosamine, *C* chondroitin, *G + C* glucosamine + chondroitin, *PBO* placebo, *NA* not available, *GI* gastrointestinal, *CV* cardiovascular, *CNS* central nervous system, *MU* musculoskeletal

### Sensitivity analysis

We also conducted sensitivity analyses for those outcomes to confirm the robustness of the results. Sensitivity analysis of sample size and methodological quality of included studies did not show any major change in view of pain, function, and stiffness (Additional file 1: Table S1).

## Discussion

In this study, we performed four individual outcome-oriented meta-analyses of randomized control trials selected on the basis of their high methodologic quality, assessing the effectiveness and safety of glucosamine, chondroitin, and the combination for the treatment of knee and/or hip OA. In our meta-analysis, the pooled effect sizes suggested that these SYSADOAs showed no significant effect on the outcome of pain, function, and stiffness compared with placebo. However, the estimates between chondroitin and placebo showed that chondroitin could alleviate pain symptoms and improve function. Compared with placebo, glucosamine proved significant effect only on the sapect of stiffness improvement. Whereas, in this head-to-head meta-analysis, the combination of glucosamine and chondroitin did not have enough evidence to be superior to placebo. There was no significant difference in the incidence of AEs and discontinuations of AEs for these SYSADOAs when compared with placebo.

Glucosamine and chondroitin are dietary supplements commonly used by those OA patents and are recommended by physician for purported analgesic and chondroprotective effects [45]. Glucosamine was considered as a water-soluble amino monosaccharide, which was one of the most abundant monosaccharides in the human body and is in high quantities in articular cartilage. Chondroitin was a major component of the extracellular matrix of articular cartilage, which played an important role in creating considerable osmotic pressure. In this way, it could provide cartilage with resistance and elasticity to resist tensile stresses during loading condition [46]. Chondroitin and glucosamine were tested in several clinical trials of osteoarthritis. In spite of the controversy surrounding the SYSADOAs, they were commonly used to control symptoms of OA in western countries. Therefore, an understanding of chondroitin and glucosamine consumption is of significance for public health.

In the previous meta-analysis, Richy and colleagues combined 7 trials of glucosamine and 8 trials of chondroitin for osteoarthritis treatment demonstrated comparable efficacies of chondroitin and glucosamine and a highly significant effectiveness of glucosamine on all involved outcomes when compared with placebo, which was contrary with our results of glucosamine and the combination therapy [47]. Collectively, their study showed that chondroitin was considered effective on pain relief, which was consistent with our finding. Additionally, a pair-wise meta-analysis of chondroitin by Monfort and colleagues suggested that chondroitin present a slight to moderate efficacy in the symptomatic treatment of OA, with an excellent safety profile [48]. The subgroup of our study covering 12 RCTs of chondroitin present that chondroitin showed significant effect in both outcome of pain and function improvement. In our study, only 4 RCTs met the criteria of combination therapy and were included in the subgroup of this meta-analysis. And glucosamine and chondroitin combination therapy failed to reduce joint pain and function improvement; this may due to original data restraints. However, this finding was similar to a least RCT publish in 2017. Roman-Blas and his colleagues indicated that chondroitin and glucosamine combination therapy failed to reduce joint pain [11]. But in the subgroup of patients with moderate-to-severe knee pain of their RCT, significant relief of joint pain with this combination therapy was observed.

Considering the reasons above, we do not oppose the use of chondroitin, although chondroitin were not recommended according to Osteoarthritis Research Society International (OARSI) guidelines published in 2014. In fact, we recommend that the future guidelines would reconsider the oral treatment option of chondroitin for the treatment of OA in the clinical feature. In terms of the aspect of safety, the current study provides valuable information to help physicians make treatment decisions for OA patients.

It was worth mentioning that a comprehensive and rigorous literature search strategy was performed in our meta-analysis, which insured that it was unlikely to miss other relevant trials. All the methods were strict inclusion and exclusion criteria to demonstrate the effectiveness and significance of our conclusions. In our meta-analysis, dosage was strictly restricted and the RCTs included should met these criteria, so the results could be comparable and reasonable. To minimize bias, studies selection, quality assessment, and data extraction were completed by two reviewers independently. What is more, several sensitivity analyses of low quality were conducted to make the results more sensible and comprehensive.

There are several limitations in this meta-analysis that need to be considered. Firstly, the quality of original data resulted in some limitation of the quality of our analysis. Secondly, in this study, there is potential publication bias. Some unpublished papers and abstracts were not taken into consideration because of unavailable data. The language might also introduce a bias. Actually, we selected only the English language. Thirdly, several specific adverse effects of interventions cannot be proven due to the inadequate reporting of adverse event data. Moreover, the numbers of RCTs between combination therapy of glucosamine and chondroitin were limited. Researches on SYSADOAs are still required due to the limitations on the quality and quantity of the available evidence.

## Conclusion

In conclusion, in accordance with our results, it can be definitively stated that oral chondroitin in recommended dosage is more effective than placebo on relieving pain and improving physical function. Compared with placebo, glucosamine showed significant effect on the outcome of stiffness. In the aspect of safety, both compounds are well tolerated. Actually, combination therapy is definitely common in clinical practice, and treatment intervention on OA patients like the combination of SYSADOAs was also usual in clinical experience. Our study would help highlight the potential role of SYSADOAs. Further studies of the glucosamine and chondroitin combination therapy need to explore the effectiveness for an accurate characterization of osteoarthritis treatment and their possible mechanism. Therefore, the above information, along with the safety profile should be conducive to clinicians in decision making.

## Additional file

Additional file 1: **Table S1.** The results of sensitivity analysis.** Figure S1.** Summary of study search and selection. RCT randomized controlled trail. **Figure S2.** Plots of bias risk. **Figure S3.** Funnel plot of effect size. (DOCX 4698 kb)

## Abbreviations

AEs: Adverse effects
DMOADs: Disease-modifying OA drugs
NSAIDs: Non-steroidal anti-inflammatory drugs
OA: Osteoarthritis
OARSI: Osteoarthritis Research Society International
RR: Relative risk
SYSADOAs: Symptomatic slow-acting drugs
