# Supplementary material for: Effectiveness and safety of glucosamine and chondroitin for the treatment of osteoarthritis: a meta-analysis of randomized controlled trials
Source: J Orthop Surg Res. 2018 Jul 6;13:170. doi: 10.1186/s13018-018-0871-5 (PMC6035477; doi:10.1186/s13018-018-0871-5)
Supplement: Supplementary file 1 — Table S1. The results of sensitivity analysis. Figure S1. Summary of study search and selection. RCT randomized controlled trail. Figure S2. Plots of bias risk. Figure S3. Funnel plot of effect size. (DOCX 4698 kb) [file 13018_2018_871_MOESM1_ESM.docx]

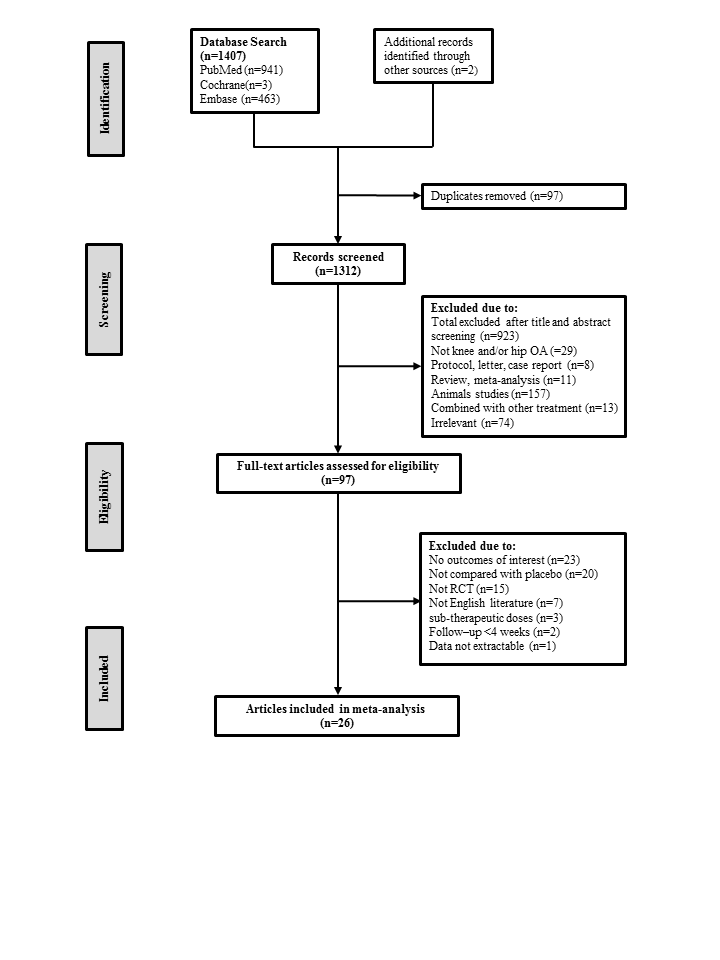


**Figure S1. Summary of study search and selection**

RCT = Randomized controlled trail


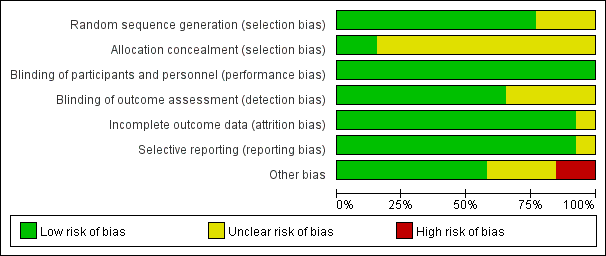


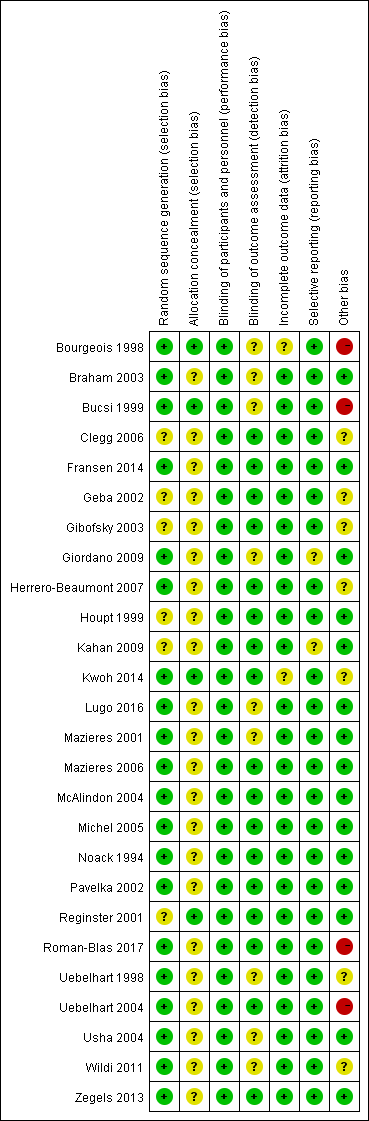


**Figure S2 Plots of bias risk**


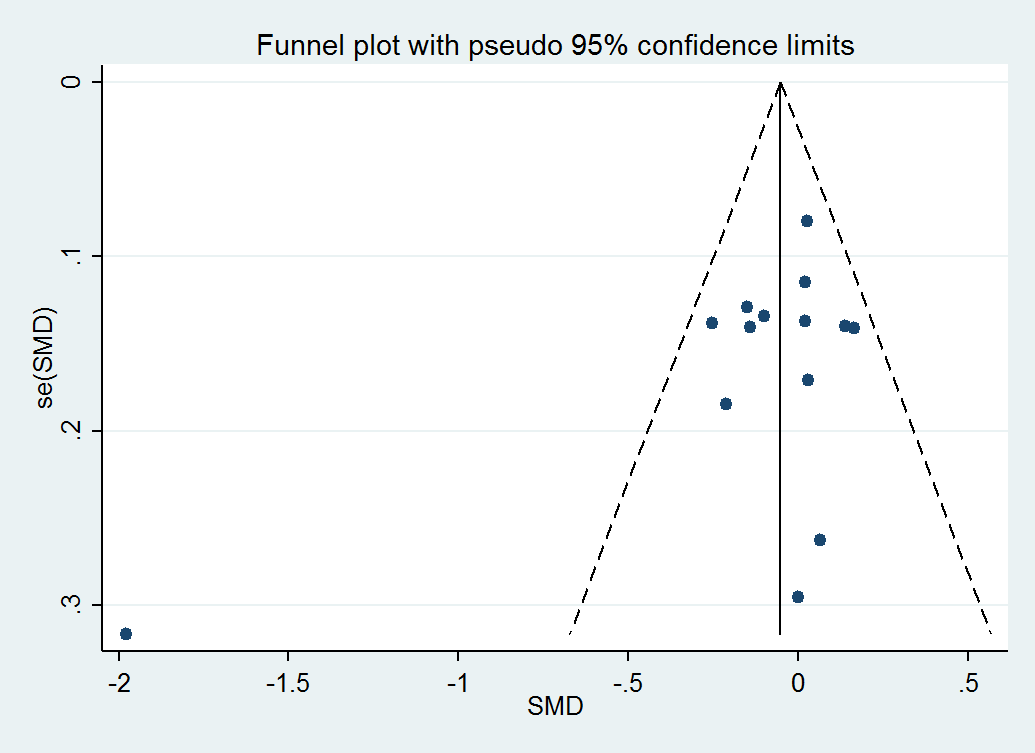


Glucosamine vs. placebo (pain)


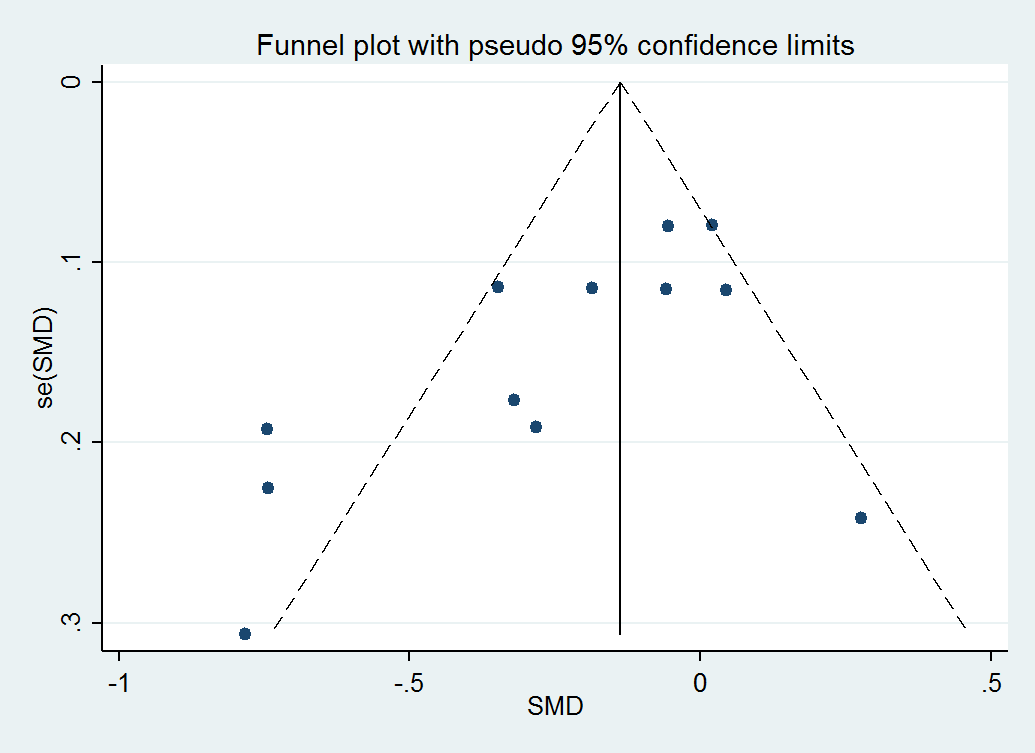


Chondroitin vs. placebo (pain)

**Figure S3 Funnel plot of effect size**

| **Table S1 The results of sensitivity analysis.** | | | |
| --- | --- | --- | --- |
| Comparison | Sensitive analysis | | |
|  | Pain | Function | Stiffness |
| Glucosamine vs Placebo | -0.02(-0.10, 0.05) | -0.06(-0.14, 0.02) | -0.05(-0.14, 0.05) |
| Chondrotin vs Placebo | -0.09(-0.16, -0.01) | -0.14(-0.25, -0.03) | 0.10(-0.03, 0.23) |
| Glucosamine + Chondrotin vs Placebo | -0.10(-0.22, 0.03) | -0.12(-0.24, 0.01) | -0.07(-0.21, 0.07) |
| Data was pooled as standard mean difference (SMD) and its related 95% CI (credibility interval) | | | |
